# Exploring early career pharmacists’ sources of stress and their coping strategies: a qualitative interview study

**DOI:** 10.1080/17482631.2026.2715826

**Published:** 2026-08-11

**Authors:** Maria B. Cooper, Sara McMillan, Fiona Kelly, Kay Dunkley, Elizabeth Hotham, Brett McDermott, Vijayaprakash Suppiah

**Affiliations:** a School of Pharmacy and Biomedical Science, College of Health, Adelaide University, SA, Australia; b School of Pharmacy and Medical Sciences, Griffith University, QLD, Australia; c Centre for Mental Health, Griffith University, QLD, Australia; d The Pharmacists’ Support Service, Melbourne, VIC, Australia; e Child and Youth Mental Health Service, Tasmania Health Service, Hobart, TAS, Australia

**Keywords:** Workplace stress, mental health, coping strategies, early career pharmacists, critical incident technique

## Abstract

**Background:**

Workplace stress is inversely correlated with job satisfaction and, when left unmanaged, can manifest as burnout. Prior research shows pharmacists under the age of thirty years and Early Career Pharmacists (ECPs) are at an especially high risk of burnout. This study aimed to: (i) explore the experiences of ECPs dealing with stress, (ii) determine the sources of stress and joy experienced by ECPs, and (iii) to explore strategies used by ECPs to reduce stress.

**Methods:**

ECPs were recruited through social media. Following informed consent, semi-structured interviews based on Critical Incident Technique (CIT) were conducted in January-February 2025. Data were analysed using CIT and reflexive thematic analysis.

**Results:**

Twenty-four ECPs (mean age 29 years; mean experience 4 years; 75% female) described 80 critical incidents. Key stressors included: 1) lack of support by management, 2) self-imposed unrealistic expectations among ECPs and 3) feeling disrespected as healthcare professionals. Participants described a need for belonging and peer connection as a coping strategy.

**Conclusion:**

The wellbeing and retention of pharmacists is paramount to an effective healthcare workforce and optimal patient care. These results have highlighted the areas requiring attention for pharmacist wellbeing, career retention and workforce sustainability.

## Introduction

The healthcare industry in Australia has rapidly seen increasing demands due to, in part, the ageing population. It has been estimated that the proportion of Australians over 65 years old will increase by almost fifty percent within the next forty years (Australian Institute of Health and Welfare. Older Australians [Internet]. Australian Institute of Health and Welfare. Australian Government; 2024 . [cited 2025 Aug 22] - please refer to the submitted text for full citation.). Numerous health risks have been associated with advanced age, such as cardiovascular and pulmonary diseases, chronic pain and mobility issues (World Health Organisation, 2024). To account for the expected increased demand in healthcare services, it is anticipated that the size of the Australian health workforce will need to increase four-fold by 2050 (Scanland & Judd, 2024).

Pharmacists play an integral role in the Australian primary healthcare system, ensuring the safe and appropriate use of medications in their communities (Pharmaceutical Society of Australia 2021). Pharmacists are also among the most accessible healthcare professionals, providing guidance on health promotion, increasing disease awareness and associated screening (Pharmaceutical Society of Australia 2021). The role of pharmacists in Australia has expanded substantially over the years; 2024 marked the ten-year anniversary of the first pilot programme involving pharmacist-led vaccination (Hava, 2024). More recently, moves to expand pharmacists’ scope of practice have culminated in the establishment of prescribing pilots for numerous acute and chronic health conditions (Queensland Government, 2025). It is anticipated that these provisions will continue to evolve in line with goals set for the future of the profession (Pharmaceutical Society of Australia, 2024).

While these developments mark significant progress for the Australian pharmacy profession, this is not withstanding concerns about workforce sustainability. Many Australian pharmacists have reported experiencing burnout, a syndrome manifested from prolonged periods of unmanaged workplace stress, (Johnston et al., 2021) which was particularly exacerbated by the COVID-19 pandemic (Johnston et al., 2021). International studies have similarly identified high levels of stress and burnout among pharmacists both prior to, and following the pandemic, with common contributing factors including excessive workload, (Johnson et al., 2014) administrative burden, (Ivanova et al., 2023) and insufficient staffing (Ivanova et al., 2024). These findings suggest the presence of a broader global workforce issue, and highlight the importance of understanding issues that impact the wellbeing of early-career professionals (Cooper et al., 2024).

Previous research has highlighted that increased workload, long working hours, managing medication shortages and patient incivility were key contributing factors towards pharmacist burnout (Bradley et al., 2023; Johnston et al., 2021). Furthermore, recent data showed that almost half of the practising Australian pharmacists surveyed had intentions to leave the profession within the next ten years, (Kamath et al., 2023) which is concerning given a current workforce shortage and a projected increased demand for pharmacist services (The Pharmacy Guild of Australia, 2024).

While reducing workplace stress is essential, supporting wellbeing also requires consideration of the positive aspects of work that allow healthcare professionals to thrive. One such concept is joy at work, which extends beyond the absence of burnout and reflects a positive workplace experience characterised by augmenting feelings of worth, fulfilment and appreciation (Jalilianhasanpour et al., 2021). While not the same as a stressful work environment, a workplace without joy can be associated with reduced productivity, increased absenteeism and employee turnover (Jalilianhasanpour et al., 2021). Understanding the factors that promote joy at work is therefore complementary to gaining a more comprehensive understanding of pharmacist wellbeing. The knowledge gained from this investigation can be used to inform strategies to support workforce development, retention, and long-term sustainability.

Early Career Pharmacists (ECPs) are those who have been working as a pharmacist for up to and including ten years (Chapman et al., 2020). This career stage is characterised by the transition from supervised training to independent clinical practice, during which pharmacists assume greater professional responsibility and accountability while continuing to develop their clinical skills, professional identity and strategies for managing complex workplace demands (Bradley et al., 2023; Noble et al., 2015). This transitional period has been recognised as an especially challenging stage for pharmacists in Australia and internationally (Bradley et al., 2023; Noble et al., 2015). A pre-COVID national survey conducted on Australian pharmacists, pharmacy interns and pharmacy students found that pharmacists under the age of thirty, and ECPs irrespective of age, were significantly more prone to stress than their older or more experienced counterparts (Chapman et al., 2020). Whilst pharmacy workplace issues were the predominant source of stress for participating pharmacists, the specific workplace factors contributing to this stress were not explored (Chapman et al., 2020). Younger age was also shown to be a contributing factor to stress and burnout among international pharmacists, with an increased risk reported in those under the age of 35 (Alhomoud & Alrasheedy, 2024; Calgan et al., 2011).

Investment in the wellbeing of ECPs is essential in developing a robust and sufficiently sized pharmacy workforce. It is crucial to retain passionate and capable ECPs, building capacity and experience in the pharmacy workforce so that they can meet the increasing demands of the Australian healthcare system and be the professional leaders of the next generation. In order to gain a deeper understanding of workplace stressors to inform better support strategies to aid wellbeing, this study aimed to: (i) explore the experiences of ECPs dealing with stress, (ii) determine the sources of stress and joy experienced by ECPs, and (iii) to explore how ECPs manage workplace stress and their coping strategies of choice.

## Materials and methods

### Population and setting

All Australian ECPs with up to ten years of general registration with the Australian Pharmacy Board were eligible to participate in this study. There were no restrictions on role, workplace setting or location where ECPs practiced. The consolidated criteria for reporting qualitative studies (COREQ) were used to guide study reporting.

### Study design and recruitment

A social media flyer was distributed to the ECP Facebook group of the Pharmaceutical Society of Australia in January 2025. At the time of writing, the group contains over fourteen thousand members from within the pharmacy profession, with group admission dependent on providing evidence of pharmacist registration in Australia. No additional outreach methods were employed. This convenience sampling approach was selected based on the target demographic of the study: young professionals aged between 22 and 35 years. Participants followed the link or scanned the QR code on the advertisement to access study information and consent forms. Interested participants then left their email addresses for further correspondence with the research team, through which an interview time was agreed upon. To ensure rigour in the data collection process, all interviews were conducted by MC. The target sample size was anticipated to be thirty participants due to the high likelihood of achieving thematic saturation; however, once this was achieved after twenty-four interviews further recruitment for the study was stopped.

The interviews utilised the Critical Incident Technique (CIT), a set of principles that allow researchers to gain an understanding of human behaviour through the discussion of defined experiences, or ‘critical incidents’ (Flanagan, 1954). Incidents are classified according to their general perception as positive or negative, or according to defining topics of each incident (Flanagan, 1954). The interview guide was informed by previous literature on the Australian pharmacy landscape, (Chapman et al., 2020) and also by prior healthcare research that utilised CIT (Holmqvist et al., 2021). The guide was piloted with one ECP in early January 2025 with no changes were made. The same interview guide was used for all participants (see Appendix).

Upon verbal consent, interviews were conducted and audio recorded via Zoom; video recording was optional. Interviews took place between January and February 2025 and averaged of 27 minutes (range: 13-47 minutes). Each interview began with several demographic questions: age, gender, state/territory of workplace, number of years registered, and time spent in current workplace. Participants were asked to discuss how they felt about being a pharmacist, and the facets of the profession that brought them the most joy and satisfaction. At interview conclusion, participants were offered an honorarium of AUD$50 gift card for their time. Participant interviews were transcribed using an AI transcription system, then manually de-identified and verified (MC). The transcripts were quality checked by two researchers (VS and JM) for accuracy. Transcripts were not returned to the participants for further comment. Reasons for not employing member checking included practical constraints, and that member checking does not traditionally yield a substantial amount of feedback (Braun & Clarke, 2006). In addition, this decision was made as a way of minimising social desirability bias.

### Data analysis

Two separate analysis techniques were utilised: CIT analysis and reflexive thematic analysis following the Braun and Clarke method (Braun & Clarke, 2006; Flanagan, 1954). Critical incidents comprised of participant recollections of exceptionally stressful situations at work. Responses about preferred wellbeing strategies were not classified as critical incidents. Familiarity with each transcript was developed through extensive reading and rereading, where emerging common patterns and themes were identified. All critical incidents were categorised as being general or specific. In this study, a specific incident involved a story detailing one interaction, either with a colleague, fellow health professional or member of the public. A general incident was related to an overall experience or feeling regarding pharmacy practice, e.g. a story relating to the overall experience of practicing unsupervised for the first time. In parallel, thematic analysis was also conducted using the qualitative research programme NVivo.

CIT and reflexive thematic analysis were used together as complementary approaches, with CIT providing insight into specific workplace experiences and reflexive thematic analysis identifying broader patterns across participant accounts. During interpretation, individual critical incidents were considered alongside the broader themes to ensure that the findings considered both personal and contextual factors that influenced pharmacist wellbeing.

At the time of study, primary researcher MC was a PhD candidate and ECP with substantial experience in community pharmacy. Although this professional background provided familiarity with the practice setting and participant experiences, this also had the potential to influence data interpretations. To enhance reflexivity, frequent supervisory team discussions were conducted in which coding decisions and emerging themes were routinely discussed. Any coding discrepancies were resolved through discussion and consensus. Although some of the participants were familiar with researcher MC or had spoken to her in passing, none had had a professional relationship with her in the five years prior to the study taking place.

### Ethics approval and consent to participate

Ethical approval for the study was obtained from the Human Research Ethics Committee at the University of South Australia (Ethics Protocol 206344). All processes of the study were conducted in accordance with the principles of the Declaration of Helsinki. All participants were provided with written information about the study, including its purpose, procedures, voluntary nature of participation, confidentiality, and the right to withdraw at any time. As the interviews were conducted online, verbal informed consent was obtained from all participants prior to data collection and was documented through audio recording at the beginning of each interview by the researcher, in accordance with ethics approval.

## Results

Twenty-four interviews were conducted. The average age of participants was 29 years, and eighteen participants were female (75%). The average time spent working as a pharmacist was four years. Most Australian states and territories were represented; there were no responses from Tasmania and the Australian Capital Territory. Most participants worked in a community setting. Additional specialities and differentiations included those in pharmacist manager positions, credentialed pharmacists to undertake medication reviews, one pharmacist who worked with medicinal cannabis, and one pharmacist undertaking an industry-related Doctor of Philosophy degree (see Table I).

**Table I.** Demographic data of interview participants (*n* = 24).

**Age**	Average age: 29.4 years (range between 23 and 42)
Gender	18 Female (75%) 6 Male (25%)
State/territory of current workplace	6 New South Wales 5 Victoria 5 Western Australia 3 Queensland 2 South Australia 1 Northern Territory
Time in profession	4.0 years (range 1 month - 9 years)
Time in workplace	Average time spent at current workplace: 2.58 years (range = 1 month -8 years)
Job description	17 community pharmacists including: 3 pharmacist managers 2 credentialed pharmacists 2 locum pharmacists 2 rural pharmacists 1 medical cannabis pharmacist 1 PhD candidate 6 hospital pharmacists 1 dual community and hospital pharmacist

Eighty critical incidents were identified, of which 32 were general and 48 were specific. Almost half (40%) of incidents centred around a consumer reaction to the participants. Incidents related to workplace culture (30%) and the increased responsibility of fully registered pharmacists (29%) were also common (Table II). These categories represent the primary critical incident sub-themes identified through the CIT analysis. Critical incidents were not restricted to a single sub-theme and could be coded to multiple categories when they reflected more than one aspect of a participant’s experience. Of the 80 critical incidents identified, 56 (70%) were coded to more than one sub-theme. Therefore, the sub-themes presented in Table II are not mutually exclusive, and frequency counts indicate the overall prevalence of each sub-theme rather than the number of unique incidents.

**Table II.** Critical incident sub-themes explained.

Theme	Explanation	General Incident *n* = 32 (%)	Specific Incident *n* = 48 (%)
Responsibility	Perceived understanding of increased authority and accountability upon full registration.	14 (43.8)	9 (18.8)
Workplace Culture	The beliefs, practices and values of a workplace that shape interactions between coworkers.	10 (31.3)	14 (29.2)
Confidence	Feelings of assuredness and perceived ability to complete tasks required of a fully registered pharmacist.	10 (31.3)	6 (12.5)
Underappreciation	Feelings of inadequate support and thanks, either by management, coworkers or the public.	6 (18.8)	4 (8.33)
Consumer Response	Verbal and physical reaction of consumers to advice or instruction.	5 (15.6)	27 (56.3)
Workload	The burden of clinical and/or administrative tasks.	5 (15.6)	2 (4.17)
Scope	Perception by coworkers, other health professionals, or members of the public.	2 (6.25)	8 (16.7)
Professionalism	Best practice, recommended guidelines and legal obligations.	2 (6.25)	7 (14.6)

Reflexive thematic analysis of the critical incidents revealed three overarching themes: 1) lack of support by management, 2) self-imposed unrealistic expectations among ECPs and 3) feeling disrespected as healthcare professionals. These overarching themes cut across multiple critical incident sub-themes, illustrating how common underlying experiences were reflected.

### Lack of support by management

Multiple ECPs felt that the stressful situations they experienced stemmed from unsupportive interactions with managerial staff. One example involved a pharmacist being questioned by a business owner when they exercised their professional judgement in refusing to supply medication to a patient without a prescription. It was implied that the pharmacist should have supplied the medication to gain the customer’s future loyalty.

*“The owner is sort of, like, having a discussion with me saying ‘why I did not supply insulin?’ and I was like “Oh... because he hasn’t been here before, and we have no records and everything, so I don’t feel comfortable giving it.” And then he sort of (said), “Oh, you can just give (it to him) ... You can just give one so we wouldn’t have so many troubles.”* - Participant 12, <1 year of experience

This example demonstrates how an ECP can feel unsupported when their professional decisions are questioned rather than endorsed, particularly at a time when they may be relying on managerial guidance. Such interactions may contribute to workplace stress by undermining the ECP’s confidence in their own judgement.

Common responses also involved experiences surrounding workload, for example instances when staffing levels were considered inadequate to manage all tasks. This was particularly evident when participants recounted experiences dating back to the COVID-19 pandemic. Additionally, participants who recalled working as sole pharmacists mentioned that they often felt guilty, and that they could not give all their patients their full attention that they may have required.

*“The worst thing, and (what) I really didn’t like, is that sometimes I feel the patient need(ed) more time.... But then looking at the baskets (full of prescriptions that need filling), I cannot give them time.”* - Participant 9, 4 years of experience

*“It’s just very challenging to wake up and then commute to work thinking about “oh, today I’m not going to have enough staff.” It’s just, that daily thought is not very positive”* - Participant 14, 4 years of experience

These accounts demonstrate how ECPs perceived inadequate staffing as a form of poor managerial support, and that they felt unable to provide sufficient support to their patients due to hiring and rostering decisions beyond their control. These circumstances may contribute to stress through ongoing tension between the ECPs’ professional responsibilities and their capacity to consistently meet patient needs.

Inability to maintain adequate staff meant significant logistical challenges for pharmacists working in geographical isolation, including rural towns. It also meant that at times, sole practicing pharmacists were often unable to take time to debrief after emotionally charged incidents with consumers. One participant described an experience in which, after being yelled at and videotaped by a consumer, she told them to leave the premises, or the police would be notified. Due to the immense workload being exacerbated by the incident, the pharmacist could not take time to process the situation.

*“I was a little bit shaky ... I only had maybe five seconds to adjust myself and take a deep breath because I still have patients waiting, and I have at least five or six orders after that that I have to pump out.”* - Participant 4, 8 years of experience

This participant described having little opportunity to debrief following an emotionally challenging interaction as ongoing workplace demands required them to immediately return to patient care. This suggests that inadequate workforce resourcing, as a result of poor managerial support, may limit ECPs’ capacity to process stressful events, potentially leading to an accumulation of emotional strain over time.

Some more newly registered pharmacists noted feeling as if their employers or managers failed to consider their lack of experience when allocating tasks and workload. One recently graduated hospital pharmacist recalled being immediately given triple the expected workload upon registration due to COVID-19-related factors. Another community pharmacist, who was employed by a large organisation, was unexpectedly rotated away from the branch where she had completed her internship immediately upon registration. Rather than establishing a client base at one pharmacy, she was abruptly scheduled to work inconsistent hours and given short notice as to where she would be working (on a rotational basis between various pharmacies). This conflicted with the ECP wanting to learn more about observing the operations of a single pharmacy, and how her contributions shaped the pharmacy over time.

One hospital ECP noted stressful situations with interpersonal relationships between managerial staff, which subsequently affected the distribution of resources across different areas of the pharmacy department. Knowledge of the perceived bias of senior staff to favour some employees meant that others felt uncomfortable approaching them with queries.

*“They (the senior staff member) had their agenda, and so that’s where some of the decision outcomes probably weren’t the best. People were very uncomfortable going into their office to talk to them.”* - Participant 2, 9 years of experience

Perceived bias in senior staff decision-making may have contributed to the disrupted communication pathways in this participant’s workplace. They described discomfort in approaching leadership, suggesting that the interpersonal dynamics among managerial staff can discourage newer and younger employees from raising concerns, thereby reinforcing the perception of unsupportive management,

### Self-imposed unrealistic expectations among ECPs

ECPs, especially those who had completed their internship more recently, described contributing to their own stress levels through unreasonably high self-expectations. They discussed the appropriateness of asking senior colleagues for help and wanting to make a good impression as a self-sufficient professional. This was something that occurred even within the first few weeks of the ECPs officially qualifying a fully registered pharmacists, able to practice autonomously for the first time.

*“The pharmacist that was working (in the role) before me was so experienced, so it’s kind of like “Okay, I’m stepping into his shoes” and they (the physicians) already have this expectation of the pharmacist.”* - Participant 5, 1 year of experience

*“Maybe once or twice a week, I could go ask (a colleague) for help, but then after that, I felt like I really need to be able to manage this on my own.”* - Participant 3, <1 year of experience

One ECP discussed how, after transitioning to a new workplace where he felt appreciated by his colleagues, he placed a large amount of undue pressure on himself to receive and maintain approval from them. This resulted in the participant becoming overworked and feeling anxious.

*“Trying to do as much as I could to kind of, I guess, impress or maintain that perception ahead of me was quite unrealistic, and it led me to kind feeling ‘Oh my god, like, I’m just—nothing I ever do is good enough, kind of thing.”* - Participant 7, 3 years of experience

Some participants noted feeling worried about making dispensing errors. Additionally, some addressed being concerned about disappointing members of their team due to not having the same amount of knowledge as their more senior colleagues. One ECP noted that being new to the profession made her feel like she wasn’t always able to assist customers and patients independently and speculated that they may have been questioning her capability as a professional, thus negatively impacting her self-confidence.

*“I know it’s okay to ask for help, but (the patient) might think that you just don’t know what you’re doing, which isn’t a nice feeling.”*- Participant 24, 2 years of experience

These accounts suggest that ECPs may internalise expectations of approval and competence in their new workplaces, leading to them placing pressure on themselves to perform beyond realistic limits. Concerns about being perceived as incompetent or inexperienced appeared to undermine participants’ confidence and contribute to anxiety, particularly if they had internalised the belief that help-seeking was a sign of professional inadequacy.

### Feeling disrespected as healthcare professionals

ECPs often did not feel listened to by members of the public, or that they experienced pushback in response to their advice or instructions. Participants reported often saying ‘no’ multiple times to convey their stance and expressed that they felt pressured or ‘guilt-tripped’. One pharmacist recalled an unreasonable request when they were asked to forge a COVID-19 vaccination certificate for a patient. Another participant discussed being asked to falsify consultation records so that a patient could obtain antibiotics for a urinary tract infection. They described feeling pressured to act in a way that went against their professional values.

*“I have had some people ask me to lie about their symptoms, you know “There was a bit of blood in the urine, but don’t put that down” or they’ll backtrack to get the drug, and I’m like “No. This is, like, a referral pathway” … and they’re like “Oh, great. So, you’re not gonna give it to me now?”* - Participant 11, 3 years of experience

Participants recalled experiences when they had to endure verbal harassment, intimidation and/or threatening behaviour from members of the public. Four participants discussed their experiences of working in pharmacies as female professionals, with two sharing how they felt concerned for their safety in the presence of violent consumers. Another reflected on how a physically larger man used intimidation tactics to pressure her into putting his prescription at the front of the line. One ECP shared that she was yelled at continuously by a patient whom she had asked to leave, who only obliged after a male bystander intervened. There were also disagreements between participants and other pharmacy staff, with the ECPs noting that factors such as their age and experience level meant that they were not being taken seriously.

*“I didn’t feel listened to (by the pharmacy tech). And I think it’s hard being young and a new pharmacist, and then you’ve got people that you’re working with that are older and a bit more experienced, so you kinda question yourself a bit.”* - Participant 20, <1 year of experience

These experiences suggest that perceived disrespect towards the ECPs was not limited to isolated incidents; rather participants described a broader pattern in which their professional authority was undermined or challenged across multiple contexts. The prominence of these experiences may reflect the characteristics of the study sample, which predominantly comprised female community pharmacists. Working in the community pharmacy setting involves frequent interactions with members of the public, which may increase exposure to situations involving conflict, emotional labour, and challenges to professional authority. As ECPs are also in the early stages of developing confidence and professional identity, repeated experiences of having their judgement or expertise challenged may influence how they perceive their role within practice.

### Sharing and community as protective factors

Most of the ECP participants discussed talking to others when they were experiencing stress in the workplace. Most participants either confided in their colleagues or friends in the pharmacy profession or debriefed with their partner/spouse or family members, with some ECPs turning to both groups for support. However, some participants explained that family members did not always understand what it meant to work as a pharmacist

*“It’s good to talk with (my partner), but sometimes we get into a fight because we have a different perspective on something.”* - Participant 13, 5 years of experience

*“As much as (my family and my partner) are there to support me ... they’re not doing pharmacy, so in the back of my mind, like, they don’t truly understand.”* - Participant 7, 3 years of experience

*Participants who reported confiding in pharmacy colleagues or university peers were grateful to speak to someone with mutual shared experience, describing that their stress was validated in the conversations they had.*

*“(My colleagues) know what happens at work, and I feel like they are just the ones who would understand.”* - Participant 22, <1 year of experience

There was also concern about placing an emotional burden on friends and colleagues, so some participants refrained from sharing as much as they may have liked.

Exercise was a commonly mentioned coping strategy used to combat stress. There were different types of exercise that varied in frequency and intensity, for example having a gym membership, taking weekly Pilates classes or daily walks either before or after work. Some participants reported being involved in team sports, such as basketball, to obtain both physical and social benefits. Other hobbies enjoyed by the ECP participants were reading, cooking, singing, listening to podcasts, sewing, dog grooming, playing video games, and taking time to rest.

*“I almost don’t have enough energy to talk about what's been happening, so then I just be silent and then just play Sudoku.”* - Participant 23, <1 year of experience

When asked about the facets of the pharmacy profession that inspired joy, most participants cited positive interactions with members of the public and being a fixture in their local communities. Forming relationships with patients and building rapport with them was an inspiring factor for many, as well as helping them to achieve positive health outcomes. In this way, developing an ongoing sense of community with their regular clientele acted as a protective factor for ECPs, reinforcing their sense of belonging as well as their purpose in the local setting.

*“It’s a tough job, but I enjoy it because I enjoy talking to the patients, helping them out... I like talking to people. I like helping people, and it’s almost a bit of, like, problem solving when I’m talking to the patients.”* - Participant 19, 3 years of experience

Collectively, these participant accounts suggest that sharing experiences and engaging in social or personal activities functioned as important protective factors against stress. Support from colleagues within the profession provided validation and a shared understanding of workplace challenges, whereas support from partners or family members was sometimes limited by a lack of understanding of the professional context. Exercise and hobbies offered opportunities for emotional recovery and disengagement from work, although some participants who used these as their preferred coping strategy also expressed reluctance to burden others with their work-related stress. Overall, the findings suggest that the effectiveness of support was shaped best through shared understanding of the realities of pharmacy practice.

## Discussion

The aim of this study was to explore the experiences of ECPs dealing with stress, determine the sources of their stress, and explore their coping strategies. To our knowledge, this study is the first globally to utilise both interviews and the critical incident technique to explore ECP experiences of workplace stress. This conclusion is supported by an unpublished systematic review of stress and wellbeing research in this population, which did not identify any studies using this combined approach (Cooper et al., 2025). The CIT allowed for further in-depth exploration of specific experiences that contributed to the stress levels of study participants. The results of this study have confirmed that Australian ECPs continue to endure workplace stress, despite the impact of the COVID-19 pandemic being notably less than it was at its height. It has also brought to attention the triggers that incite feelings of anxiety and disheartenment in this cohort.

Prevalent themes among the critical incidents captured in this study were that the ECPs felt unsupported by workforce management, and that stress often stemmed from exposure to poor working conditions, including high workloads and insufficient staffing. These findings align with job demand-resources theory, which suggests that high job demands such as workload and time pressure, combined with limited workplace resources can contribute to reduced employee wellbeing (Schaufeli, 2017). Workforce shortages remain an ongoing challenge within Australian pharmacy, with difficulties recruiting and retaining pharmacists reported across the profession (Kamath et al., 2023; The Pharmacy Guild of Australia, 2023). Similar concerns have been identified internationally where insufficient staffing and unsustainable workloads have been reported as significant contributors to pharmacist stress.

Responsibility was a prominent theme among the critical incidents identified in this study. The transition from internship to full registration may also represent a period of role transition stress, as ECPs move from a supervised learning environment to autonomous practice. Challenges experienced during this period may therefore not only increased workload demands, but also the broader process of socialisation into professional practice (Jalilianhasanpour et al., 2021; Noble et al., 2015). Accredited training programmes are mandatory for intern pharmacist in Australia to support to the transition to an autonomous health professional (Australian Health Practitioner Regulation Agency, 2021). While these programmes offer substantial support in broadening interns’ clinical knowledge and consultation skills, a much smaller focus is placed on the development of leadership and managerial skills, which are potentially inadequate in meeting the demands of the workplace of these newly qualified pharmacists. Responsibility was a prominent theme among the critical incidents identified in this study.

Anxiety surrounding the ‘jump’ in responsibility between internship and full registration could be mitigated through the integration of leadership and management training on other settings, for example in the workplace, or in tertiary education. Alternatively, another approach would be a restructuring of community pharmacy role classification similar to hospital pharmacy, in which responsibility is increased in small increments based on experience level (Pharmaceutical Society of Australia, 2015). Although practical and systemic differences between community and hospital pharmacy practice may present implementation challenges, such an approach may provide a framework for ECPs to gradually build confidence and develop their skills.

The increased responsibility associated with full registration was described as overwhelming by ECPs in this study, particularly in relation to being solely accountable for clinical and dispensing decisions that could potentially impact patient wellbeing. The ECPs reported that they experienced joy through positive interactions with patients and seeing improvements in health outcomes due to their care, while negative experiences such as confrontational encounters or dispensing errors, were associated with considerable stress. International literature supports the presence of stress in ECPs, with American pharmacy residents (a position similar to Australian pharmacy interns) identifying time pressures, excessive workload and the fear of errors as key stressors (Zinurova & DeHart, 2018). These findings provide a broader context for understanding the experienced described by participants in the present study. Concerns regarding workload and dispensing errors have been discussed in Australia since at least 1999 (Peterson et al., 1999). However, there remained limited research specifically examining the emotional impact of dispensing errors on Australian pharmacists, despite evidence from the United Kingdom and New Zealand reporting that these incidents elicited feelings of guilt, fear, shame and burnout (Edwards, 2021; Johnson et al., 2014). Further research in the Australian context may be warranted to better understand the emotional impact of medication errors on ECPs and inform strategies that support adjustment to increased professional responsibility.

Participants in this study reported that they sought out professional peers for emotional support. This highlights the fulfilment that participants gain in feeling understood by others, particularly regarding their professional role and the reasoning behind their actions. Recent research has reinforced the relationship between emotional coping strategies (e.g. spending time with friends, colleagues, family and pets) with reduced workplace stress in Australian pharmacists (Shahin et al., 2023). Additionally, avoidant coping responses such as social withdrawal and excessive alcohol consumption had a strong positive correlation with increased stress (Shahin et al., 2023). While some coping strategies may provide short-term relief, those that limit opportunities for support-seeking or addressing workplace issues may have different longer-term implications for wellbeing. These results, and the desire for community voiced by current study participants, emphasise the need for interventions that can facilitate socialisation in this professional group. This may be particularly important for ECPs who are practicing in geographically remote areas, or those prone to professional isolation by working as a sole practitioner. Research on the benefits and accessibility of virtual peer support conducted among Australian medical professionals, have highlighted the desire of many study participants to feel part of a community (Cooper et al., 2025). Avenues such as this may be helpful in creating a non-judgemental social network for ECPs in need of reassurance and a safe space to talk through workplace issues.

Even within the ECP populations, generational differences may have influenced how participants perceived and responded to stressful workplace situations. The age range of participants spanned those at the older end of the millennial generation through to those born after 2000. However, with a sample of twenty-four ECPs, there was insufficient evidence to draw firm conclusions regarding age-related differences in perceptions of workplace expectations and demands. The demographic profile of study participants suggests a predominance of community pharmacists and female participants, which may have influenced thematic outcomes. Yet, this is reflective of the demographic makeup of the Australian pharmacist workforce; almost two-third of pharmacists identify as female, (Australian Health Practitioner Regulation Agency, 2023) and approximately 53% of pharmacists work in community pharmacy compared to just 8% of the workforce in the hospital sector (The Pharmacy Guild of Australia, 2024). As identified previously, the findings related to intimidation, harassment and safety concerns have significant implications for workforce wellbeing and retention, considering pharmacy being a female majority profession (Bakken et al., 2021; Bissell et al., 2021; Peterson et al., 2011). These experiences highlight the importance of ensuring psychological safety and appropriate organisational responses to inappropriate consumer behaviour. Employers must make system-level changes such as having violence preventative strategies, trauma counselling and reporting of violent incidences to enhance safety in workplaces.

The study recruitment process may have introduced the possibility of self-selection bias, particularly attracting participants who may have been more engaged, dissatisfied or interested in topics related to wellbeing. However, this method was chosen to allow for wider exposure to many Australian ECPs. As such, results may not be reflective of the views of ECPs outside of the group, or those who are less active on social media. Therefore, the findings should be interpreted considering the characteristics of study participants. Additionally, study data collection relied solely on the recollection of incidents from ECPs, meaning that it is subject to recall bias (Pannucci & Wilkins, 2011). Few participants in this study were from rural or remote areas, suggesting that issues pertaining to this group of ECPs may be underrepresented in the study. However, a strength of this study was that the interview method of data collection allowed for study participants to share their lived experiences of workplace stress in greater depth than would normally be collected through a survey. The intimate environment of a one-to-one interview also meant that study participants were able to be open in their discussion, something that may have not occurred in a focus group scenario due to fear of judgement.

## Conclusion

Australia’s aging population is growing, highlighting a need for a larger healthcare workforce, inclusive of pharmacists. This study aimed to explore the experiences of ECPs in relation to stress, workplace joy and coping strategies. Participants described sources of joy relayed to positive interactions with patients and the local community, as well as observing patient wellbeing improve following their interventions. However, they also described feeling under-supported in the workplace perceived that their professional role was not adequately recognised and feeling overwhelmed by the transition from provisional to general registration. ECPs debriefed from stressful situations by talking to others, such as a colleague, partner or spouse. These findings suggest that peer-based support strategies, including virtual peer networks, may warrant consideration to support wellbeing during the early stages of professional practice.

## Supplementary Material

Supplementary Material Interview CoreQ Checklist.pdf

## Data Availability

The participants of this study did not give written consent for their data to be shared publicly, hence supporting data is not available due to the public due to the sensitive nature of the data.

## Appendix A: interview guide

### **Part one: introduction and permissions**

- Thank the participants for their involvement in the project.
- Explain the purpose of the research (to gain a deeper understanding of the lived experiences of early career pharmacists, particularly those that make them feel pressured or stressed).
- Assure the participant that their data will be de-identified so that no one can link them to a particular response.
- Obtain permission to record the interview
- Advise that the topic of the interview may bring up some feelings of anxiety etc. Assure the participant that they are able to exit the interview at any time. Mention some immediate wellbeing resources that can be accessed e.g. BeyondBlue, Pharmacists’ Support Service.
- Ascertain permission to call or email in the days following the interview to check on the participant’s emotional wellbeing.
- Encourage the participant to speak freely and assure that there is no judgement in this study environment.

### **Part Two: demographic questions**

- What is your age and gender identity?
- What state of Australia do you work in?
- What is your role as a pharmacist? How many hours per week do you work? Could you describe what a typical week looks like for you? Eg. are you at the same pharmacy all the time?
- How many years have you been registered as a pharmacist?
- How many years have you been working at your current workplace?
- If the participant is working as a locum, how long have they been in that locum kind of role?

### **Part three: understanding the participant experience**

The main purpose of our interview today is for you to share some stories about particular experiences that you’ve had working as a pharmacist. How do you generally feel about being a pharmacist?

** added 16/01/25 - What are the things about being a pharmacist that bring you joy?

Think of the most recent time you experienced an event in the pharmacy that made you feel stressed or pressured. Be as specific as you can be, from the start of the incident until after it was over.

If a participant can’t recall any specific event, prompt and ask about a more general experience.

Tell me when you last felt stressed or pressured ...

1. Briefly describe the incident/experience

a. Where were you?

b. How did it start?

2. How did it make you feel?

a. What were you thinking during the incident/experience?

3. Were there other people involved in the situation?

a. If so, what did they do?

What did you (participant) do?

4. What was the outcome of this incident/experience?

a. No outcome? What do you mean by that?

5. How did this incident/experience affect you?

6. What happened after the incident/experience?

7. How do you think that this incident/experience could have been avoided?

Have you experienced another incident like this that you would like to talk about?

- If yes, repeat questions 1 to 7

- Continue for as many incidents as the participant wants to discuss.

What do you like to do outside of work to support your wellbeing?

- Is there a particular person you who talk to when you need emotional support?

To conclude: Is there anything else of relevance that you would like to discuss today?
